# Association between Diabetes Mellitus and Oral Health Status in Patients with Cardiovascular Diseases: A Nationwide Population-Based Study

**DOI:** 10.3390/ijerph18094889

**Published:** 2021-05-04

**Authors:** Su-Jin Han, Youn-Jung Son, Bo-Hwan Kim

**Affiliations:** 1 Department of Dental Hygiene, College of Health Science, Gachon University, (21936) 191 Hambakmoero, Yeonsu-gu, Incheon 21936, Korea; sjhan@gachon.ac.kr; 2 Red Cross College of Nursing, Chung-Ang University, (06974) 84 Heukseok-ro, Dongjak-gu, Seoul 06974, Korea; yjson@cau.ac.kr; 3 College of Nursing, Gachon University, (21936) 191 Hambakmoero, Yeonsu-gu, Incheon 21936, Korea

**Keywords:** cardiovascular diseases, diabetes mellitus, oral health, periodontitis

## Abstract

Diabetes mellitus (DM) can lead to poor oral health. However, oral health among diabetic patients with cardiovascular diseases (CVDs) is scarcely studied. This study aimed to elucidate the prevalence of oral health complications and the relationship between DM and oral health status in diabetic patients with CVDs. This retrospective nationwide cross-sectional study evaluated 3495 patients aged ≥40 years with CVD, with DM (*n* = 847) and without DM (*n* = 2648). The participant’s characteristics between the two groups were compared using the Chi-square test and t-test. Logistic regression analyses were performed to identify associations between DM and oral health status. The prevalence of periodontitis (54.3% vs. 43.2%) and <20 number of remaining teeth (30.9% vs. 22.8%) was significantly higher in the DM than in the non-DM group. In the multivariate regression analysis, the incidence of periodontitis was 1.4 times higher in the DM group than in the non-DM after adjusting for confounders; however, the number of remaining teeth and active caries were not associated with DM. In conclusion, the oral health status of patients with coexisting CVD and DM should be assessed closely and actively. Healthcare professionals should provide accessible dental care services and develop strategies to improve patients’ oral health.

## 1. Introduction

In addition to being a major cause of mortality globally, cardiovascular diseases (CVDs) are also the principal contributors to decreased quality of life [1,2,3]. According to the Global Burden of Disease Study, 2017, the largest number of deaths among noncommunicable diseases (17.8 million) were attributed to CVD [1]. Among all cases of CVD, such as angina and myocardial infarction, approximately 32.2% were associated with diabetes mellitus (DM). Moreover, because CVD is a major cause of mortality in people with DM, they accounted for approximately half of all deaths that occurred globally between 2007 to 2017 [4]. In the United States National Health Interview surveys from 2000 to 2009, individuals diagnosed with both CVD and DM reportedly included approximately 26% of women and 31% of men [5]. The combination of ischemic heart disease and DM is one of the most adverse conditions that lead to a significant increase in cardiovascular complications and mortality [6]. Therefore, healthcare professionals must consider the adverse effects and comorbid impact of CVD and DM.

Oral health is closely associated with CVD and DM, specifically in terms of the incidence of oral diseases, such as periodontal disease and dental caries or their progression [7,8]. Periodontal disease is an inflammatory disorder affecting the tissues surrounding the teeth. Periodontitis is a more severe and chronic condition that can result in tooth loss. Moreover, chronic inflammatory periodontal diseases such as periodontitis are well-known risk factors for DM and CVD, which hints at a bidirectional link [7]. Indeed, periodontitis is based on the chronic inflammatory model seen in CVD and DM [7]. Periodontal pathogenic microorganisms have been detected in disparate tissues and organs of the cardiovascular system [9,10,11], exhibiting endocarditis, myocarditis, pericarditis, and atherosclerotic lesions [12]. In addition, knowledge and attitudes or awareness of oral health in people with diabetes are closely associated with oral health complications [13,14]. Therefore, oral health problems should be assessed in close association with coexisting DM and CVD.

Among people with CVD, those with DM might have a higher risk of incident periodontitis than people without DM [15]. In fact, after the five major DM complications, namely, CVD, peripheral vascular disease, retinopathy, neuropathy, and nephropathy [16], aggressive periodontitis was reported as the sixth major complication [17]. In patients with unceasingly uncontrolled hyperglycemia, systemic inflammatory or immune responses may cause periodontal disease and dental caries [18], leading to a progressive cardiovascular risk [19].

Similar to the relationship between DM and oral health [15,18,20] or the relationship between CVD and oral health [19,21,22], various studies have suggested associations between DM or CVD and oral health. However, only a few studies have examined the oral health status in CVD patients with or without DM [23]. Further, South Korea lacks research on oral health in middle-aged or older adults [24,25]. Moreover, studies on oral health considering coexisting diabetes and cardiovascular disease, which have a higher prevalence than other diseases, are sporadic. Therefore, using the latest Korea National Health and Nutrition Examination Survey (KNHANES) VII dataset from 2016 to 2018, we aimed to identify the prevalence of oral health problems and the relationship between DM and oral health status among diabetic patients with CVD.

## 2. Materials and Methods

### 2.1. Design, Sample, and Setting

This study used data acquired from the KNHANES VII (Korea Centers for Disease Control and Prevention (KCDC) [26]), conducted by the KCDC from 2016 to 2018. The KNHANES is a nationally representative, cross-sectional study designed to assess the health and nutritional status of the noninstitutionalized Korean population. It extracts 23 households from 192 primary sample units as a probability sample every year, followed by surveying individuals aged ≥1 year. The sampling protocol was designed to involve a complex, stratified, multistage, clustered probability survey of a representative sample of the noninstitutionalized civilian population in South Korea. A detailed description of the sampling protocol is described in the KNHANES VII guidelines [27].

**Figure 1:**
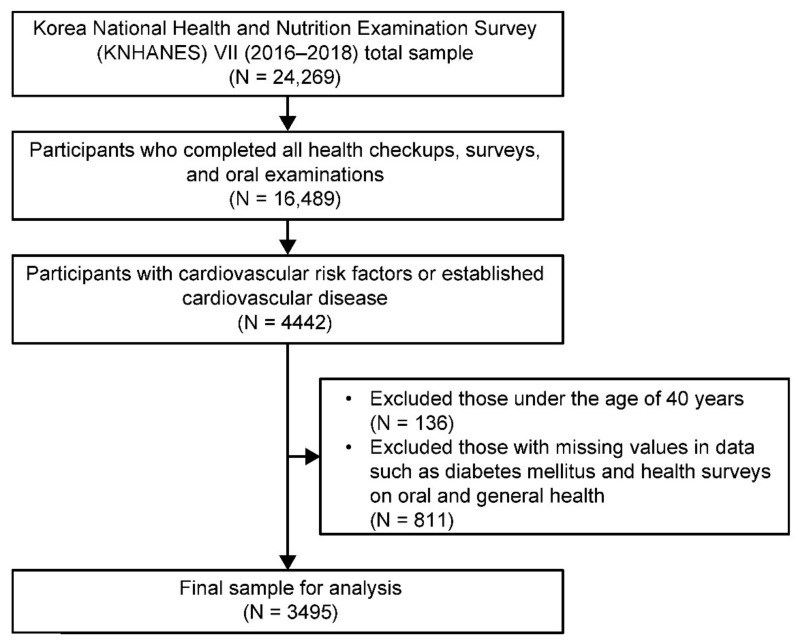
Flow diagram of the study participants.

A total of 16,489 out of 24,269 participants from the KNHANES VII completed all health checkups and surveys, including oral examinations. From these, we identified 4442 individuals with two major cardiovascular risk factors (hypertension and dyslipidemia) or established CVD (stroke, myocardial infarction, and angina pectoris). We included people with hypertension and dyslipidemia because these are two major contributing risk factors of CVD and because of the additive adverse impact on the vascular endothelium which results in enhanced atherosclerosis, leading to CVD a few years later [28]. We excluded 811 participants with missing data such as DM values and health surveys on oral and general health. We also excluded 136 individuals under the age of 40 because mortality and prevalence of cardiovascular risk factors such as obesity, hypertension, diabetes mellitus, and dyslipidemia are known to increase more rapidly among individuals over 40 or those who are middle-aged or more [29,30]. Finally, we used data from 3495 participants for the analysis (Figure 1).

### 2.2. Measurement

The survey consisted of a health interview, health examination, and nutrition survey, which obtained a range of information about health status, health behavior, socioeconomic demographics, and laboratory tests. All indicators were nominal categorical variables, and they followed the KNHANES VII guidelines [27].

#### 2.2.1. Diabetes Mellitus

In this study, DM was defined as fasting plasma glucose ≥ 126 mg/dL, self-reported diagnosed diabetes, current use of oral hypoglycemic agents, and insulin use, except for type I DM. DM was categorized as “yes” or “no.”

#### 2.2.2. Oral Health Status

Oral health status was determined by assessing periodontitis, active caries, and the number of remaining teeth. Periodontal tissue of the participants was evaluated using the Community Periodontal Index, following the World Health Organization (WHO) guidelines [31]. The Community Periodontal Index codes were classified as follows: 0, normal periodontal tissue; 1, presence of gingival bleeding; 2, presence of calculus; 3, presence of a 4–5-mm pocket; and 4, presence of a ≥6-mm pocket. In this study, periodontitis was confirmed when a participant presented with codes “3” or “4” during the examination and if there were one or more periodontal pockets with a depth of ≥4 mm. Furthermore, active caries were classified as “yes” or “no”, based on the presence of active caries or lesions on the teeth, and the number of remaining teeth was categorized into ≥20 teeth and <20 teeth according to the number of teeth present in the mouth. Trained examiners performed oral examinations according to the WHO guidelines [31].

#### 2.2.3. Other Potential Confounding Variables

We adjusted for confounding variables related to diabetes as correction variables to identify the association with oral health status. We selected sociodemographic, clinical, general health behavior, and oral health care variables as potentially confounding variables.

Sociodemographic characteristics included age, sex, living together, education, job, and household income [20,32]. Education was classified into three groups based on the Korean education system: below the primary school, middle–high school, and college or higher. Household income was subdivided into quartile groups based on monthly income: <25% (the lowest quartile group), 25–49%, 50–74%, and ≥75% (the highest quartile group).

Data on general health behavior variables such as smoking status, drinking status, and subjective health status were collected [20]. Smoking status was classified into “yes” (currently smoking and those who smoked >100 cigarettes during their lifetime) and “no” (nonsmoker or past smoker) according to current smoking habits [33]. Participants were grouped into “drinker” and “nondrinker” based on their lifetime drinking experience.

To analyze the oral healthcare parameters, questions regarding tooth brushing frequency, use of oral care products, dental clinic visits, and dental checkups were asked [20]. Daily tooth brushing frequency was categorized into once or less, twice, and three or more times. The use of dental floss or interdental brush was categorized as “yes” or “no”. Dental clinic visits to receive treatment for oral health problems, including examination and prevention, were classified as “yes” or “no”, based on participant’s response to the following question: “Have you ever visited the dental clinic in the past 1 year?” Dental checkups for regular examination of teeth and gums were classified as “yes” or “no” according to the experience of undergoing a regular oral examination to check the oral health status in the past year.

Clinical characteristics were derived from health interviews and health examination data. The health interview confirmed whether the participant had been diagnosed by a doctor for diseases. The health examination data included body mass index, waist circumference, blood pressure, and laboratory data [20,32,34]. Body mass index was calculated by dividing the weight (kg) by the square of height (m^2^). Body mass index was then classified into five groups according to the WHO Asia-Pacific Guideline [35]: <18.5 kg/m^2^, 18.5–22.9 kg/m^2^, 23.0–24.9 kg/m^2^, 25.0–29.9 kg/m^2^, and ≥30.0 kg/m^2^. Laboratory data included the participants’ blood analysis results for total cholesterol, triglycerides, high-sensitivity C-reactive protein, blood urea nitrogen, serum creatinine, and hemoglobin [20,21,32].

### 2.3. Data Analysis

The sampling design of the KNHANES VII implemented a complex multistage, stratified, and unequally weighted or clustered selection of sample units. All data were weighted for statistical analyses. Regarding oral examination, only some survey zones were used as the survey conditions for the KNHANES VII (2016–2018); therefore, a separate weight was assigned by integrating 3 years. In this study, a plan file was created by applying an integrated weight, including an oral examination. After assessing the normality of the distribution of continuous variables using the Kolmogorov−Smirnov test, all continuous data were expressed as mean ± standard error. Categorical data were expressed as numbers and percentages. To compare the characteristics of participants between the DM and non-DM groups, we used the Chi-square test and independent t-test. We applied logistic regression analyses to identify the association between DM and each oral health status (periodontitis, number of remaining teeth, and active caries). Model 1 was unadjusted. In Model 2, variables that were confirmed to be related to diabetes in the univariate analysis were added to adjust for correction variables. All analyses were conducted using IBM SPSS Statistics for Windows, version 25.0 (IBM Corp., Armonk, NY, USA). Statistical significance was considered for two-tailed *p*-values < 0.05.

The minimum sample size of people with missing teeth and periodontitis to satisfy the study requirements was estimated to be between 254 and 1340 adults. The association between missing teeth/periodontitis and CVD with DM/myocardial infarction, a part of CVD, was estimated using the following parameters: 5% of the standard error, 95% of power, 95% of the confidence interval (CI), and an odds ratio (OR) of at least 1.78 or 1.28 to be detected for logistic regression analysis [22,23]. The actual number of participants was larger than the minimum required as determined by these parameters. Power curves were calculated using G*Power 3.1.9.7 [36], and the required minimum sample size needed at a range of power levels was indicated considering the logistic regression test [37].

### 2.4. Ethical Considerations and Data Collection

This study was approved by the institutional review board of Gachon University (No.: 1044396-202004-HR-085-01) and conducted in accordance with the principles of the Declaration of Helsinki. Participants were informed regarding voluntary consent provision and written informed consent was obtained before the survey. Trained interviewers conducted face-to-face interviews with the participants, and all participants were physically examined by trained staff.

## 3. Results

### 3.1. General Characteristics of People with and without DM Combined with CVD

Table 1 shows a comparison between the sociodemographic characteristics and general health behaviors of people with CVD in the DM and non-DM groups. Older age (*p* < 0.001), male sex (*p* = 0.026), living alone (*p* < 0.001), lower education level (*p* < 0.001), no job (*p* = 0.014), and lower household income (*p* < 0.001) were significantly associated with DM. In addition, regarding general health behavior, the DM group included a significantly higher prevalence of “drinkers” (*p* = 0.019) and those with poor subjective health status (*p* < 0.001) than the non-DM group. Regarding oral health care, the DM group had a significantly higher number of people with a lower tooth brushing frequency (*p* < 0.001), no dental clinic visits (*p* = 0.037), and no dental checkups (*p* < 0.001) compared with the non-DM group.

### 3.2. Clinical Characteristics of People with and without DM Combined with CVD

Table 2 shows a comparison of clinical characteristics between people with CVD in the DM and non-DM groups. The DM group had a significantly higher prevalence of hypertension (*p* = 0.001), dyslipidemia (*p* < 0.001), stroke (*p* = 0.033), myocardial infarction (*p* = 0.008), and renal failure (*p* = 0.006). In addition, a higher obesity rate (*p* = 0.035), larger waist circumference (*p* < 0.001), decreased diastolic blood pressure (*p* < 0.001), low total cholesterol (*p* < 0.001), high blood urea nitrogen (*p* = 0.002), high serum creatinine (*p* = 0.001), and low hemoglobin (*p* = 0.005) were associated with an increased incidence of CVD.

### 3.3. Oral Health Status of People with and without DM with CVD

Regarding oral health status, the DM group had significantly more prevalence of participants with periodontitis (54.3% vs. 43.2%, *p* < 0.001) and <20 remaining teeth (30.9% vs. 22.8%, *p* < 0.001) than the non-DM group. There was no statistically significant difference in the presence of active carious lesions between the groups (Table 3).

### 3.4. Logistic Regression Analysis of the Association between DM and Oral Health Status in CVD

As shown in Table 4, univariate logistic regression revealed that people with DM had an increased risk of periodontitis (OR = 1.529, 95% CI: 1.272–1.837) and a higher risk of having a lower number of remaining teeth (OR = 1.530, 95% CI: 1.240–1.886) compared with those in the non-DM group. Multiple logistic regression revealed that people with DM had an increased risk of periodontitis (adjusted OR = 1.395, 95% CI: 1.150–1.693) than those without DM after adjusting for confounding variables. However, the number of remaining teeth and active caries parameters were not associated with DM after adjusting for covariates in the multivariate regression analysis.

## 4. Discussion

Many previous studies evaluating the relationship between DM and CVD with oral health status were conducted at primary levels or using small sample sizes. Even though CVD accompanied by DM could seriously affect oral health [7], we could not locate any relevant studies. Therefore, this is the first report on the relationship between CVD coexisting with DM and oral health status using recent national survey data. Our key findings were that, among people with CVD, the prevalence of periodontitis (54.3% vs. 43.2%) and <20 remaining teeth (30.9% vs. 22.8%) was significantly higher among people with DM than in those without DM. Furthermore, in the multivariate regression analysis, the incidence of periodontitis was 1.4 times higher in the DM group than in the non-DM group after adjusting for confounding variables.

In a previous study with a nationwide sample of the general population in the 2012 KNHANES of adults over 30 years old [20], the prevalence of periodontitis was 43.7% in adults with DM compared to 25% in those without DM. Furthermore, a study involving multiple datasets extracted from the National Health and Nutrition Examination Survey (NHANES) 2009–2014 in the USA found a moderate-severe periodontitis prevalence of 36.4% [38]. The results from our study showed that oral health problems among people with CVD were 54.3% more prevalent in those with DM than those without DM. Our results showed that periodontitis was highly prevalent among patients with coexisting CVD and DM.

In addition, our study revealed that the prevalence of <20 remaining teeth was higher among CVD patients with DM (30.9%) than among CVD patients without DM. These results are consistent with a Japanese study which revealed that the number of missing teeth among people with CVD with DM was significantly higher than that among people with CVD without DM [23]. Notably, the number of missing teeth in CVD patients with DM might suggest irreversible end-stage periodontitis [23]. Therefore, health workers should regularly assess oral health status in CVD patients with DM.

The univariate logistic regression analysis results showed a 1.56- and 1.51-fold increase in the prevalence of periodontitis and the number of remaining teeth, respectively, among CVD patients with DM than those without DM. However, after adjusting for confounding variables, periodontitis showed a 1.41-fold increased prevalence among CVD patients with DM. These findings contradict those of a recently reported Japanese study [23] in which the number of missing teeth in the DM group was higher than that in the non-DM group [23]. However, they reported no significant difference between the groups in terms of aggravated periodontitis, such as the incidence of edentulism, probing pocket depth, clinical attachment level, or the incidence of bleeding on probing [23]. Based on our results, this difference may be justified because the Japanese study only included men aged 69–80 years, and the total number of participants (*n* = 239) in that study was substantially lower than our study. Additionally, older people tend to lose teeth more easily than younger people [39], and men with DM showed a 23% association with tooth loss [40]. Therefore, future studies should be conducted using large national surveys from other countries to identify the influence of oral health status, such as periodontitis and tooth loss, on people with coexisting CVD and DM. We recommend a prospective cohort study that investigates oral disease and subsequent CVD in people with type 2 diabetes. In addition, it is necessary to identify the association between diabetes and periodontitis in CVD through a systematic review and metaregression analysis of prospective longitudinal studies. Healthcare providers must be made aware of oral health-related quality of life and oral health changes among CVD patients with DM.

Our study findings should be interpreted in light of the following few limitations. First, the cross-sectional design precludes conclusions about causal relationships; thus, further prospective studies and interventional trials should be undertaken to establish a causal association between DM and oral health status with oral health-related quality of life. Second, even though both CVD and DM have a bidirectional effect on periodontitis, we could not wholly exclude participants who repeatedly experienced oral health problems before diagnosing CVD or DM. Oral health status was evaluated by asking a single question and conducting oral examinations. This prohibited us from evaluating the duration and prevalence of oral health problems. Thus, these results should be interpreted carefully. Third, because the effect of periodontitis due to medication adherence and dental treatment for CVD patients with DM was not excluded, the association between periodontal inflammation and DM may be underestimated. Fourth, periodontal status was assessed using the Community Periodontal Index. Although the Community Periodontal Index is widely used to evaluate periodontal treatment needs in a community setting, it can underestimate the prevalence of periodontitis because of the use of 10 index teeth and the possibility of pseudo pockets [41]. Nevertheless, this is a widely used method in numerous epidemiological studies [42,43,44]. Lastly, because these results were from a nationwide population-based study from only one Asian country, the findings may not be generalizable. More studies across various countries, including Europe and North America, are required in order to obtain strong evidence.

## 5. Conclusions

CVD patients with DM can have compromised oral health. This study was the first to report a higher prevalence of oral health problems such as periodontitis and <20 remaining teeth among CVD patients with DM. We recommend that healthcare professionals should provide accessible dental care services to CVD patients with DM and develop strategies to improve their oral health.

## Figures and Tables

**Table 1:** General characteristics of people with CVD in the DM and non-DM groups.

Variables	Total	Non-DM Group	DM Group	*p*-Value
	*n* = 3495	*n* = 2648	*n* = 847	
**Socio-demographics**				
Age (years), *n* (%)				<0.001 ^#^
40–49	320 (8.9)	268 (9.7)	52 (6.1)	
50–59	851 (25.1)	694 (26.8)	157 (19.3)	
60–69	1127 (32.2)	850 (31.9)	277 (33.1)	
70–79	937 (26.6)	644 (24.4)	293 (33.5)	
≥80	260 (7.3)	192 (7.1)	68 (8.1)	
Sex, *n* (%)				0.026 ^#^
Male	1556 (42.9)	1148 (41.7)	408 (47.0)	
Female	1939 (57.1)	1500 (58.3)	439 (53.0)	
Living status, *n* (%)				<0.001 ^#^
Alone	647 (15.4)	456 (14.1)	191 (20.0)	
Together	2848 (84.6)	2192 (85.9)	656 (80.0)	
Education, *n* (%)				<0.001 ^#^
≤Elementary	1375 (38.8)	997 (37.1)	378 (44.6)	
Middle-high school	1457 (41.6)	1099 (41.6)	358 (41.4)	
≥College	663 (19.6)	552 (21.3)	111 (14.1)	
Job, *n* (%)				0.014 ^#^
Yes	1773 (49.1)	1383 (50.5)	390 (44.8)	
No	1722 (50.9)	1265 (49.5)	457 (55.2)	
Household income ^a^, *n* (%)				<0.001 ^#^
<25%	1113 (31.1)	778 (28.7)	335 (38.8)	
25–49%	913 (25.9)	703 (26.2)	210 (25.0)	
50–74%	732 (21.2)	558 (21.5)	174 (20.4)	
≥75%	737 (21.7)	609 (23.6)	128 (15.8)	
**General health behaviors**				
Smoking status, *n* (%)				0.067 ^#^
Non-smoker/past smoker	2973 (85.6)	2268 (86.3)	705 (83.4)	
Current smoker	522 (14.4)	380 (13.7)	142 (16.6)	
Drinking status, *n* (%)				0.019 ^#^
Nondrinker	604 (16.8)	432 (15.9)	172 (19.8)	
Drinker	2891 (83.2)	2216 (84.1)	675 (80.2)	
Subjective health status, *n* (%)				<0.001 ^#^
Good	635 (18.3)	517 (19.7)	118 (13.7)	
Moderate	1795 (52.2)	1396 (53.5)	399 (48.2)	
Bad	1065 (29.4)	735 (26.8)	330 (38.1)	
**Oral health care**				
Tooth-brushing ^b^, *n* (%)				<0.001 ^#^
≤1	499 (14.0)	349 (12.8)	150 (17.7)	
2	1503 (42.4)	1113 (41.1)	390 (46.5)	
≥3	1493 (43.6)	1186 (46.0)	307 (35.8)	
Usage of floss or interdental brush, *n* (%)				0.068 ^#^
No	2639 (74.9)	1966 (74.0)	673 (77.8)	
Yes	856 (25.1)	682 (26.0)	174 (22.2)	
Dental clinic visit, *n* (%)				0.037 ^#^
No	1329 (38.3)	986 (37.3)	343 (41.6)	
Yes	2166 (61.7)	1662 (62.7)	504 (58.4)	
Dental checkup, *n* (%)				<0.001 ^#^
No	2325 (66.3)	1713 (64.3)	612 (72.9)	
Yes	1170 (33.7)	935 (35.7)	235 (27.1)	

Categorical values are presented as unweighted number (weighted %). # Chi-square test. ^a^ Quartile groups of household income. ^b^ Frequency/day of tooth brushing. DM, diabetes mellitus.

**Table 2:** Clinical characteristics and oral health care in people with and without DM with CVD.

Variables	Total	Non-DM Group	DM Group	*p* -Value
	*n* = 3495	*n* = 2648	*n* = 847	
Hypertension, *n*(%)				0.001 ^#^
No	958 (28.8)	777 (30.5)	181 (23.1)	
Yes	2537 (71.2)	1871 (69.5)	666 (76.9)	
Dyslipidemia, *n* (%)				<0.001 ^#^
No	1558 (43.1)	1225 (45.1)	333 (36.4)	
Yes	1937 (56.9)	1423 (54.9)	514 (63.6)	
Stroke, *n* (%)				0.023 ^#^
No	3260 (93.4)	2487 (94.0)	773 (91.4)	
Yes	235 (6.6)	161 (6.0)	74 (8.6)	
Myocardial infarction, *n* (%)				0.012 ^#^
No	3381 (97.0)	2571 (97.4)	810 (95.5)	
Yes	114 (3.0)	77 (2.6)	37 (4.5)	
Renal failure, *n* (%)				0.016 ^#^
No	3473 (99.5)	2636 (99.7)	837 (99.1)	
Yes	22 (0.5)	12 (0.3)	10 (0.9)	
BMI (kg/m^2^), *n* (%)				0.017 ^#^
<18.5	45 (1.4)	37 (1.4)	8 (1.2)	
18.5–22.9	926 (28.0)	734 (29.2)	192 (23.9)	
23.0–24.9	918 (26.0)	702 (26.4)	216 (24.6)	
25.0–29.9	1362 (38.7)	996 (37.1)	366 (43.9)	
≥30.0	244 (6.0)	179 (5.9)	65 (6.4)	
Waist circumference (cm)	85.97 ± 0.21	85.30 ± 0.23	88.19 ± 0.37	<0.001 ^†^
Systolic blood pressure (mmHg)	126.53 ± 0.39	126.75 ± 0.43	125.84 ± 0.72	0.245 ^†^
Diastolic blood pressure (mmHg)	75.76 ± 0.23	76.78 ± 0.26	72.41 ± 0.40	<0.001 ^†^
Total cholesterol (mg/dL)	184.80 ± 0.80	190.55 ± 0.89	165.93 ± 1.50	<0.001 ^†^
Triglyceride (mg/dL)	151.38 ± 2.56	149.66 ± 2.93	157.02 ± 4.20	0.130 ^†^
hs-CRP (mg/dL)	1.29 ± 0.04	1.29 ± 0.05	1.29 ± 0.08	0.981 ^†^
Blood urea nitrogen (mg/dL)	16.44 ± 0.12	16.27 ± 0.13	16.99 ± 0.21	0.002 ^†^
Serum creatinine (mg/dL)	0.85 ± 0.01	0.84 ± 0.01	0.89 ± 0.02	0.001 ^†^
Hemoglobin (g/dL)	13.87 ± 0.03	13.92 ± 0.04	13.72 ± 0.07	0.006 ^†^

Categorical data are presented as unweighted numbers (weighted %). Continuous variables are estimated as mean ± standard error. # Chi-square test. † Student’s *t*-test. DM, diabetes mellitus; BMI, body mass index; hs-CRP, high-sensitivity C-reactive protein.

**Table 3:** Oral health status in people with and without DM with CVD.

Variables	Total	Non-DM Group	DM Group	*p* -Value
	*n* = 3495	*n* = 2648	*n* = 847	
Periodontitis				<0.001 ^#^
No	1877 (54.2)	1491 (56.8)	386 (45.7)	
Yes	1618 (45.8)	1157 (43.2)	461 (54.3)	
Number of remaining teeth				<0.001 ^#^
≥20	2636 (75.3)	2054 (77.2)	582 (69.1)	
<20	859 (24.7)	594 (22.8)	265 (30.9)	
Active caries				0.221 ^#^
No	2574 (74.8)	1961 (75.3)	613 (72.9)	
Yes	921 (25.2)	687 (24.7)	234 (27.1)	

Categorical data are presented as unweighted numbers (weighted %). ^#^ Results were obtained using the Chi-square test. DM, diabetes mellitus.

**Table 4:** Association of DM with oral health status in people with CVD by logistic regression analysis.

DM	Periodontitis		Number of Remaining Teeth		Active Caries	
	OR (95% CI)		OR (95% CI)		OR (95% CI)	
	Model 1	Model 2	Model 1	Model 2	Model 1	Model 2
Yes	1.561 ** (1.300–1.874)	1.406 * (1.160–1.705)	1.511 ** (1.220–1.858)	1.184 (0.934–1.510)	1.136 (0.926–1.393)	1.082 (0.874–1.341)
No	1	1	1	1	1	1

Response variables: periodontitis, number of remaining teeth, active caries. Explanatory variable: DM. Model 1 was not adjusted. Model 2 was adjusted for age, sex, living together, education, job, household income, drinking status, subjective health status, hypertension, dyslipidemia, stroke, myocardial infarction, renal failure, body mass index, waist circumference, total cholesterol, blood urea nitrogen, serum creatinine, hemoglobin, diastolic pressure, tooth brushing frequency, dental clinic visit, and dental checkup within a year. * *p* < 0.01, ** *p* < 0.001. DM, diabetes mellitus; OR, odds ratio; CI, confidence interval.

## Data Availability

Publicly available datasets were analyzed in this study. This data can be found here: https://knhanes.kdca.go.kr/knhanes/sub03/sub03_02_05.do (accessed on 13 May 2020).
